# Molecular characterization of *Klebsiella pneumoniae* isolated from different clinical sources in Eastern and Western Regions of Saudi Arabia Tertiary Hospitals

**DOI:** 10.7717/peerj.21653

**Published:** 2026-09-02

**Authors:** Zahra ALBrahim, Nasreldin Elhadi, Faye Aldehalan, Asif A. Jiman-Fatani, Dalya M. Attallah

**Affiliations:** 1 Department of Clinical Microbiology and Immunology, Faculty of Medicine, King Abdul Aziz University, Jeddah, Saudi Arabia; 2 Department of Clinical Laboratory Sciences, College of Applied Medical Sciences, Imam Abdulrahman Bin Faisal University, Dammam, Saudi Arabia; 3 Clinical and Molecular Microbiology Laboratories, King Abdulaziz University Hospital, Jeddah, Saudi Arabia

**Keywords:** Epidemiology, ERIC-PCR, Carbapenemase-resistant *Klebsiella pneumoniae*, Saudi Arabia, Multidrug-resistant bacteria

## Abstract

**Introduction:**

The prevalence of extensively drug-resistant (XDR) and multidrug-resistant (MDR) strains worldwide has limited the ability of antimicrobial therapy to treat severe bacterial infections. The emergence and rapid global dissemination of carbapenemase-resistant *Klebsiella pneumoniae* (CRKP) strains occluded the therapeutic use of carbapenems, the last resort of β-lactams. In Saudi Arabia, major outbreaks of carbapenemase-producing Enterobacteriaceae have been reported, with approximately a 90% prevalence rate of CRKP strains. In this study, we aimed to delineate the prevalence and resistance patterns of the CRKP isolates in clinical samples from tertiary hospitals in the Eastern and Western regions of Saudi Arabia.

**Methods:**

CRKP isolates were tested for carbapenem resistance using the disc diffusion method. Molecular screening and identification of these isolates for carbapenemase genes and conducted molecular genotyping using Enterobacterial Repetitive Intergenic Consensus Polymerase Chain Reaction (ERIC-PCR) DNA fingerprinting to map clonal relatedness.

**Results:**

CRKP was identified in 112 clinical samples, all showing resistance to standard antimicrobials and at least one carbapenem. Molecular investigation of these CRKP isolates revealed a high epidemiological prevalence of *Klebsiella pneumoniae* carbapenemase (KPC) (91.1%) along with Oxacillinase-48 (OXA-48) (a type of class D β-lactamase) (92.9%), while New Delhi metallo-β-lactamase (NDM) was only observed in the Western region. ERIC-PCR fingerprinting was able to group strains into 12 distinct clusters with more than 95% genetic similarity.

**Conclusion:**

This may indicate the attainment of an endemic state of the KPC genes, highlighting the need for an urgent establishment of stringent containment policies. These findings indicate the broad distribution of carbapenemase-producing *Klebsiella pneumoniae* in both regions, highlighting the urgent need for enhanced surveillance and strengthened infection control strategies. The molecular genotyping using ERIC-PCR showed a high level of genetic similarity among isolates, with limited genetic variability.

## Introduction

The emergence of antimicrobial resistance (AMR) has become a global public health crisis, primarily driven by the misuse and overuse of antibiotics (Zaman et al., 2017). Among multidrug-resistant (MDR) pathogens, *Klebsiella pneumoniae* is a highly virulent Gram-negative bacterium responsible for severe hospital-acquired infections, including bacteremia, pneumonia, urinary tract infections, and meningitis (Basak, Singh & Rajurkar, 2016; World Health Organization, 2024a; World Health Organization, 2024b). *K. pneumoniae* is commonly referred to as a “superbug” because of its increasing resistance to carbapenems, which are considered the last line of β-lactam antibiotics (Hammoudi Halat & Ayoub Moubareck, 2020; Lan et al., 2021; Mohd Asri et al., 2021; Zowawi et al., 2014).

The global spread of carbapenem-resistant *K. pneumoniae* (CRKP) has escalated due to the emergence of high-risk clones; however, their clinical characteristics vary depending on local conditions and regional geography (Hansen, 2021; Wyres & Holt, 2022). New Delhi metallo-β-lactamase (NDM), *K. pneumoniae* carbapenemase (KPC), and Oxacillinase-48 (OXA-48) carbapenemases mainly cover the majority of widespread CRKP strains, which have caused sporadic outbreaks or progressed to an endemic state. The rapid spread of carbapenemases is attributed to the horizontal transfer of these resistance genes into high-risk CRKP clones, including ST11, ST258, and ST512 (Karampatakis, Tsergouli & Behzadi, 2023; Logan & Weinstein, 2017; Perlaza-Jiménez et al., 2021; Tesfa et al., 2022).

*K. pneumoniae* strains were assumed to belong to two separate groups: hypervirulent strains (cause severe infections but are curable) or multidrug-resistant strains (difficult to treat but not aggressive). An increasing number of isolates exhibit overlapping hypervirulent and multidrug-resistant genetic traits. Recent studies have demonstrated the emergence of hybrid lineages that are difficult to treat, posing a significant clinical and epidemiological concern (Karampatakis, Tsergouli & Behzadi, 2023; Lan et al., 2021; Wyres & Holt, 2022).

In Saudi Arabia, NDM-type carbapenemase-producing Enterobacterales (CPE) have become endemic, and reports from the Arabian Peninsula suggest that NDM genes are the most prevalent (Al-Zahrani & Alsiri, 2018; Alraddadi et al., 2022; Shibl et al., 2013). The 2017–2018 reports of the Global Antimicrobial Resistance Surveillance System (GLASS) described a 10–30% prevalence rate of CPE in Saudi Arabia. The report also showed that the isolates of CRKP reported from various regions of Saudi Arabia showed a tremendous increase during 2017–2018 (WHO, 2018). The inappropriate use of antibiotics, lack of awareness, and travel history of populations from the Asian continent are the major risk factors that have led to the increased prevalence of CPE in the population of Arab countries (Alotaibi, 2019).

The epidemiology of carbapenem-resistant *K. pneumoniae* shows variation from region to region. Regional variation in prevalence, dominant carbapenemase types, and circulating clonal lineages differs from one region to another (Hansen, 2021; Wyres & Holt, 2022). In Saudi Arabia, KPC, OXA-48, and NDM-producing strains have been reported with varying regional dominance (Pitout, Nordmann & Poirel, 2015; Zowawi et al., 2014). Recent reports from Gulf countries indicate an increase in the prevalence of CRKP, posing a threat to healthcare settings in the Gulf region. In Saudi Arabia, it was observed that the blaOXA-48-like and blaNDM genes were the most common resistance genes in CRKP isolates, along with the emergence of high-risk strains of CRKP carrying several resistance genes (Al-Zahrani et al., 2024; Alharbi et al., 2026; Alkhelaiwi et al., 2026). The same findings were observed in Qatar and other Gulf Cooperation Council member states, where the genomic screening showed the widespread presence of OXA-48-like- and NDM-producing CRKP strains in these regions (Tsui et al., 2023; Al Fadhli et al., 2023).

This highlights local transmission dynamics and the importance of region-specific surveillance. Understanding these regional differences is essential for interpreting molecular epidemiology findings and control strategies.

Given the limited epidemiological data from the Eastern and Western Provinces of Saudi Arabia, this study aims to assess the prevalence and molecular characteristics of CRKP isolates collected from tertiary hospitals in Jeddah and Al-Khobar. The study further investigates the resistance profiles and genotypic diversity of these isolates using Enterobacterial Repetitive Intergenic Consensus Polymerase Chain Reaction (ERIC-PCR) to enhance understanding of regional dissemination patterns and support targeted infection control strategies.

## Methods

### Collection of bacterial isolates

All clinical isolates (112) used in this study were collected from June 2020 through December 2021 from two different tertiary hospitals (*n* = 57 strains) and King Abdulaziz University Hospital (KAUH) in the Western Province of Saudi Arabia (*n* = 55 strains). It was ensured that there was only one isolate per patient/ sample event was included to ensure no duplicate samples were analyzed. Only one non-duplicate CRKP isolate per patient was included in the study to avoid overrepresentation of persistent or repeated isolates from the same individual. This approach ensured independent observations for molecular and epidemiological analysis. Samples were collected from male and female patients across different age groups to cover the entire demographic spectrum. Clinical isolates were extracted from a wide range of infection sites to avoid ambiguity. This study was approved by the Research Ethics Committee (REC) in King Abdulaziz University with NCBE Registration No. (HA-02-J-008).

### Bacterial strains and DNA extraction

Clinical isolates of *K. pneumoniae* were grown on MacConkey agar plates overnight at 37 °C to obtain single colonies. Next, the isolated colonies were grown on nutrient agar plates and incubated overnight at 37 °C. Single colonies from nutrient agar plates were sub-cultured on Luria Bertani (LB) broth and incubated in an orbital shaker for 18 h at 37 °C. DNA was extracted using boiling techniques as described (Elhadi, 2016). The boiling method was chosen for DNA extraction because it enables rapid processing of a large number of isolates with minimal reagent requirements while yielding DNA of adequate quality and purity for Polymerase Chain Reaction (PCR) amplification. Given that downstream applications were limited to PCR-based detection of resistance genes and ERIC-PCR typing, this approach was considered appropriate and efficient. Briefly, one mL of incubated LB broth was transferred into a 1.5 mL tube, and pellets for all bacterial isolates were obtained at 1,000×g for 2 min. Pellets were resuspended in 500 µL of sterilized distilled water and boiled at 100 °C for 15 min. The boiled isolates were kept at −20 °C immediately for PCR.

### Carbapenemase gene amplification

The diluted cell lysates were subjected to PCR in a concentration of 12% per sample along with 50% of master mix (MOLEQULE-ON, Auckland, New Zealand), 8% each of forward and reverse marker gene primers (MOLEQULE-ON) (Table S1), and 30% of sterile distilled water. A total PCR reaction volume of 25 µL was set up in the thermocycler.

PCR protocols were designed according to the primers and amplicons. The annealing temperature was determined based on primer *T*_m_, and the extension time was determined based on the expected size of the amplicon. Thus, there were slight variations in the PCR protocol for each carbapenemase gene. Based on these criteria, the PCR protocols below were designed specifically for each carbapenemase gene (Table S2). The amplified product was verified for the presence of that class of carbapenemase in a particular clinical isolate, and a positive control was included for screening of each gene using in-house positive control strains previously (Abdalhamid et al., 2017). The PCR-amplified products were subjected to gel electrophoresis on a 1% (w/v) agarose gel. The molecular weight of the amplified product was estimated using a 100 bp DNA ladder (MOLEQULE-ON). The agarose gels were visualized and photographed using a gel documentation system (G:BOX; Syngene). A representative agarose gel image demonstrating the PCR amplification results, including the DNA ladder (marker), positive control, negative control, and representative OXA gene-positive test isolates, was provided as Fig. S1.

### Antimicrobial susceptibility testing

All clinical isolates were tested for antimicrobial susceptibility using the Kirby-Bauer disc diffusion method and automated systems (VITEK; MicroScan), following the Clinical and Laboratory Standards Institute (CLSI) guidelines (Candan & Aksoz, 2015). Inoculum turbidity was adjusted to a 0.5 McFarland standard and evenly spread on Mueller–Hinton agar plates. Antibiotic discs were applied, and plates were incubated at 37 °C for 18–24 h, after which inhibition zone diameters were measured. Minimum inhibitory concentrations (MICs) were determined for carbapenems using automated systems and interpreted according to CLSI breakpoints.

For non-carbapenem antibiotics, susceptibility results were expressed as categorical interpretations (Susceptible, Intermediate, Resistant), derived from either disc diffusion zone diameters or MIC values, and interpreted according to CLSI guidelines (Table S3). These interpretations were derived from disc diffusion zone diameters and/or MIC values obtained from automated systems. The percentage susceptibility values presented in the results reflect these categorical interpretations. *E. coli* ATCC 25922 and *Pseudomonas aeruginosa* ATCC 27853 were used as control strains.

Both phenotypic (disc diffusion and MIC determination) and genotypic (PCR-based detection of carbapenemase genes) approaches were employed to ensure comprehensive and accurate characterization of antimicrobial resistance.

### Molecular genotyping by ERIC-PCR

ERIC-PCR was performed using ERIC2 primer (5′-AAGTAAGTGACTGGGGTGAGCG-3′) as described (Alsultan & Elhadi, 2022; Elhadi, 2018). Briefly, a 20 µL PCR reaction mixture was prepared, consisting of 3 µL of extracted DNA template, 10 µL PCR master mix (MOLEQULE-ON, New Zealand), 2 µL ERIC2 primer, and 5 µL nuclease-free deionized water per reaction. Thermal cycling conditions included an initial denaturation at 94 °C for 4 min, followed by 35 cycles of denaturation (94 °C, 45 s), annealing (52 °C, 1 min), and extension (65 °C, 8 min), with a final extension at 65 °C for 10 min. Amplified PCR products were analyzed on 1% agarose gels stained with ethidium bromide and visualized using the G:BOX documentation system.

### ERIC fingerprinting analysis

The analysis of DNA fingerprints generated by ERIC-PCR was performed using GelJ software to investigate the DNA fingerprint (Heras et al., 2015). The unweighted average pair group method (UPGMA) and the Cosine coefficient were used to examine the genetic relationship among strains. The analysis of DNA fingerprints was performed at a position tolerance and optimization value of 1%. Discrimination of ERIC fingerprints were performed with a cut-off of 95% to differentiate the ERIC fingerprint types of the investigated strains.

### Statistical analysis

Data were coded and entered using IBM SPSS Statistics for macOS, Version 27. Descriptive statistics, including frequency, percentage, median, and interquartile range (IQR), were used to summarize demographic and clinical characteristics.

## Results

### Demographic distribution of clinical isolates

A total of 112 clinical isolates of *K. pneumoniae* were collected from tertiary hospitals in Jeddah (Western Province) and Al-Khobar (Eastern Province). The distribution of samples was nearly equal between the two cities, with 55 isolates (49.1%) from Jeddah and 57 isolates (50.9%) from Al-Khobar. The study included an equal number of male (*n* = 56) and female (*n* = 56) patients. Most isolates (54.5%) were obtained from patients aged between 45 and 80 years. Urine and blood were the most common specimen types, together accounting for 47.3% of all isolates (Table 1).

**Table 1:** Demographic data and sources of the clinical isolates (*N* = 112).

**Parameters**	**Level**	**Frequency** **(n)**	**Percentage** **(%)**
Gender	Male	56	50.0
	Female	56	50.0
Age (years)	<45	27	24.1
	45–80	61	54.5
	>80	24	21.4
City	Al-Khobar	57	50.9
	Jeddah	55	49.1
Type of specimen	Sputum	9	8.0
	Blood	24	21.4
	Urine	29	25.9
	Respiratory	2	1.8
	Transtracheal	21	18.8
	Peritoneal fluid	3	2.7
	Cerebrospinal fluid	2	1.8
	Tissue	7	6.3
	Wound	15	13.4

### Prevalence of Carbapenemase-producing K. pneumoniae

All 112 isolates demonstrated resistance to at least one carbapenem, confirming their classification as CRKP. Molecular screening using PCR revealed a high prevalence of both KPC and OXA–48 carbapenemase genes, detected in 91.1% and 92.9% of isolates, respectively. Notably, the NDM gene was found exclusively in isolates from Jeddah, suggesting potential regional variation in gene distribution. Other carbapenemase genes, including IMI (3.6%) and IMP (0.9%), were detected at low frequencies (Tables 2 and 3). No amplification was observed for SME, SIM, VIM, SPM, or GIM genes. Age-wise analysis showed that the majority of isolates harbouring carbapenemase genes were from patients aged 45–80 years (Table 4).

**Table 2:** Profiles of different classes of carbapenemases (*N* = 112).

**Genes**	**Positive**	
	Frequency (n)	Percentage (%)
OXA–48	104	92.90
NDM	18	16.10
KPC	102	91.10
IMP	1	0.90
VIM	0	0.00
NMC	0	0.00
SIM	0	0.00
GIM	0	0.00
SPM	0	0.00
SME	0	0.00
GES	0	0.00
IMI	4	3.60

**Table 3:** Prevalence of *K. pneumoniae* strains harbouring multiple carbapenemase genes (*N* = 112).

**Carbapenemase genes**	**Jeddah**	**Al-Khobar**	**Overall**
	n (%)	n (%)	n (%)
KPC	3 (2.7)	1 (0.9)	4 (3.6)
OXA–48	0 (0)	10 (8.9)	10 (8.9)
NDM + KPC	4 (3.6)	0 (0)	4 (3.6)
OXA–48 + KPC	31 (27.7)	46 (41.1)	77 (68.8)
OXA–48 + KPC + IMI	3 (2.7)	0 (0)	3 (2.7)
OXA–48 + KPC + NDM	12 (10.7)	0 (0)	12 (10.7)
OXA–48 + KPC + NDM + IMI	1 (0.9)	0 (0)	1 (0.9)
OXA–48 + KPC + NDM + IMP	1 (0.9)	0 (0)	1 (0.9)

**Table 4:** Age-wise prevalence distribution of isolates for carbapenemase genes.

**Genes**	**Result**	**<45 years**		**45–80 years**		**>80 years**	
		***n***	**(%)**	***n***	**(%)**	***n***	**(%)**
OXA–48	Positive	24	88.90	57	93.40	23	95.80
NDM	Positive	7	25.90	6	9.80	5	20.80
KPC	Positive	25	92.60	55	90.20	22	91.70
IMP	Positive	0	0.00	0	0.00	1	4.20
VIM	Positive	0	0.00	0	0.00	0	0.00
NMC	Positive	0	0.00	0	0.00	0	0.00
SIM	Positive	0	0.00	0	0.00	0	0.00
GIM	Positive	0	0.00	0	0.00	0	0.00
SPM	Positive	0	0.00	0	0.00	0	0.00
SME	Positive	0	0.00	0	0.00	0	0.00
GES	Positive	0	0.00	0	0.00	0	0.00
IMI	Positive	1	3.70	3	4.90	0	0.00

**Notes.**

n frequency % Percentage

Approximately 90% proportion of isolates co-harbored both KPC and OXA-48 carbapenemase genes. This unusually high rate of co-carriage represents a significant epidemiological finding, as the coexistence of multiple carbapenemase genes within a single isolate may enhance resistance profiles and limit therapeutic options.

### Detailed epidemiology of carbapenemase genes

To better understand the clinical distribution of carbapenemase genes among CRKP isolates, Table 3 presents individual-level data collected from patients in Jeddah and Al-Khobar, respectively. These include demographics, sample source, gene positivity, and Ambler classification for each isolate.

### Antimicrobial susceptibility patterns

Antimicrobial susceptibility testing revealed widespread resistance among the CRKP isolates. High resistance was observed against β-lactams, fluoroquinolones, and aminoglycosides. The isolates showed the lowest resistance to colistin and tigecycline. A full summary of susceptibility patterns for each antimicrobial agent is presented in Table 5.

**Table 5:** Antimicrobial-susceptibility pattern of *K. pneumoniae* isolates.

**Antimicrobial used**	**Susceptible** ** (S)** **n (%)**	**Intermediate (I)** **n (%)**	**Resistant (R)** **n (%)**
Aztreonam (ATM)	8 (7.1)	3 (2.7)	101 (90.2)
Ceftazidime (CAZ)	0 (0)	1 (0.9)	111 (99.1)
Cefotaxime (CTX)	0 (0)	1 (0.9)	111 (99.1)
Ceftriaxone (CRO)	0 (0)	0 (0)	112 (100)
Ciprofloxacin (CIP)	0 (0)	1 (0.9)	111 (99.1)
Cefepime (FEP)	0 (0)	1 (0.9)	111 (99.1)
gentamicin (CN)	13 (11.6)	0 (0)	99 (88.4)
Kanamycin (K)	3 (2.7)	2 (1.8)	107 (95.5)
Levofloxacin (LEV)	0 (0)	2 (1.8)	110 (98.2)
Imipenem (IPM)	0 (0)	2 (1.8)	110 (98.2)
Norfloxacin (NOR)	0 (0)	2 (1.8)	110 (98.2)
Nitrofurantoin (F)	0 (0)	3 (2.7)	109 (97.3)
Trimethoprim-sulfamethoxazole (SXT)	6 (5.4)	3 (2.7)	103 (92)
Piperacillin-tazobactam (TZP)	0 (0)	0 (0)	112 (100)
Ticarcillin (TIC)	0 (0)	0 (0)	112 (100)

**Notes.**

n frequency S Susceptible I Intermediate R Resistant

Quantitative MIC analysis demonstrated elevated carbapenem resistance levels among all CRKP isolates. For imipenem, MIC values ranged from 8 to ≥16 µg/mL. Thirty-eight isolates (33.9%) demonstrated an MIC of 8 µg/mL, while 74 isolates (66.1%) exhibited MIC values of ≥16 µg/mL. The calculated MIC50 and MIC90 for imipenem were both ≥16 µg/mL. For meropenem, all 112 isolates (100%) showed MIC values of ≥16 µg/mL, indicating uniformly high-level resistance. The distribution of MIC values for carbapenems is summarized in Table 6.

**Table 6:** Distribution of MIC values for carbapenems.

**Antibiotic**	**MIC (µg/mL)**	**Number of isolates (n)**	**Percentage (%)**
Imipenem	8	38	33.9
Imipenem	≥16	74	66.1
Meropenem	≥16	112	100

### Molecular typing of CRKP isolates

**Figure 1:**
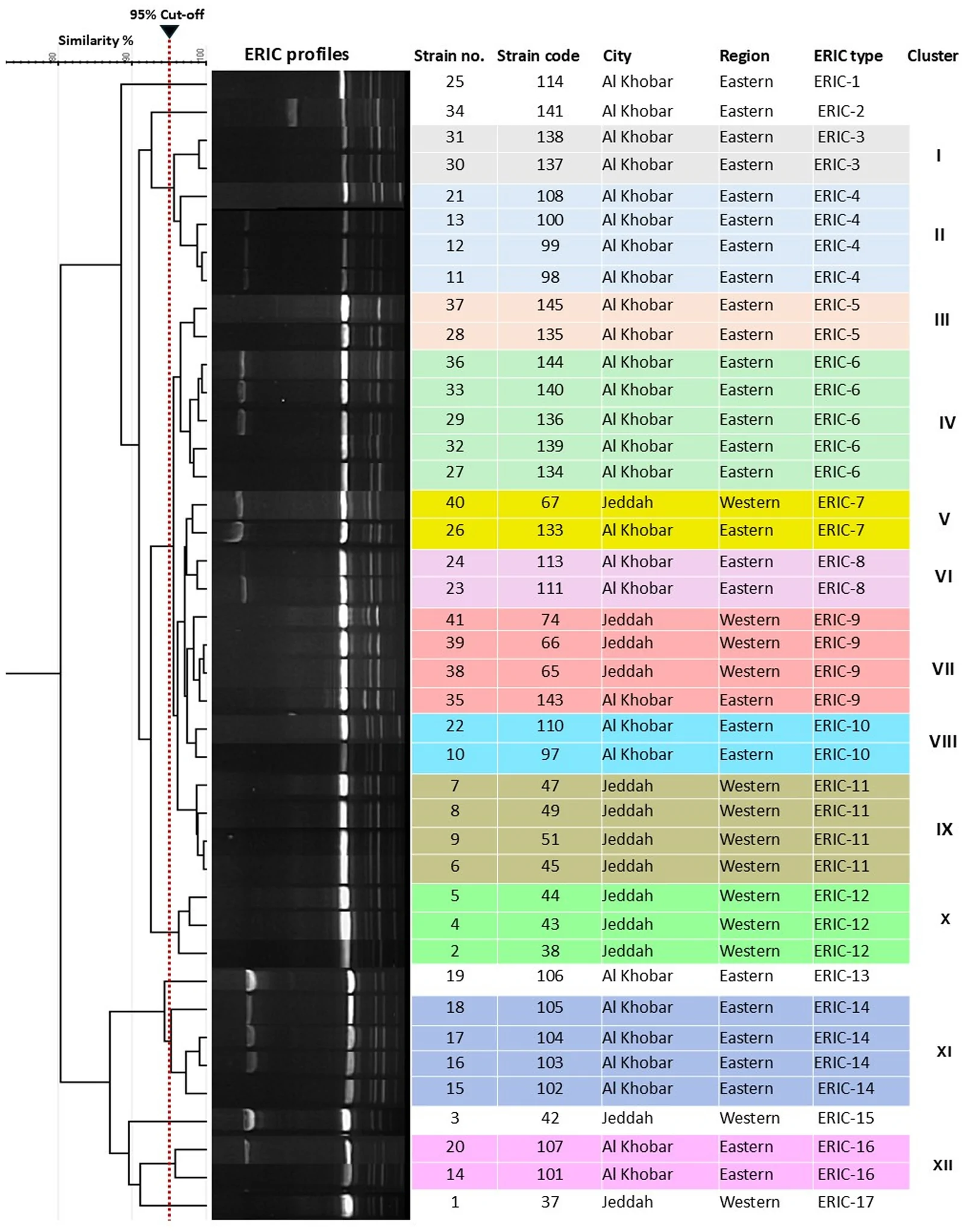
Dendrogram for the ERIC-PCR DNA fingerprints generated using the ERIC2 primer. Data calculated using the Cosine coefficient and UPGMA. The top bar indicates the similarity percentage, and the arrow at the top shows the clusters formed at a 95% cut-off.

In this study, all 112 strains were typed by ERIC-PCR. The fingerprints were obtained using the ERIC2 primer, generating six to eight bands ranging in size from 180 to 2,400 bp, and all investigated strains shared 700 bp DNA fragments, as shown in Fig. S2. Among the 112 strains typed by ERIC-PCR, 41 representative strains were analyzed using GelJ software and grouped the ERIC-PCR fingerprints into 12 clusters (I to XII) at a cut-off value of 95%, as shown in Fig. 1. Two strains isolated in Western and Eastern regions were clustered together in cluster V with 98% genetic similarity (Fig. 1). Similarly, three strains isolated from different clinical specimens in Jeddah, in the Western region, were grouped in cluster VII, with one strain isolated from a 66-year-old male patient’s blood specimen in Al Khobar, in the Eastern region of Saudi Arabia, with 98% genetic similarity (Fig. 1). Interestingly, the dendrogram generated from ERIC-PCR fingerprinting of 41 strains revealed that strains positive for the NDM gene were grouped in clusters VII and IX, and all these strains were isolated from different clinical sources in Jeddah in the Western region (Fig. 1).

## Discussion

The global emergence of carbapenemase-producing *Enterobacteriaceae* has raised significant public health concerns (Alsultan & Elhadi, 2022). Genetic history plays a critical role in understanding their pathogenesis and drug resistance. Understanding the genetic background and its relationship with pathogenesis is essential for developing efficient control strategies (Elhadi, 2018). Various species of *Enterobacteriaceae* have developed carbapenem resistance, with *K. pneumoniae* being the most prevalent strain in Saudi Arabia (Alotaibi, 2019).

The extensive use of antimicrobials in healthcare settings has contributed to the rise of resistance. Carbapenems, being the last line of defense, have shown reduced effectiveness (Heras et al., 2015). The Riyadh region and southern provinces report the highest number of cases (Brisse et al., 2009; Lai et al., 2017). However, clinical data from other regions are limited. This study aimed to address this gap by analyzing clinical and microbiological data from Jeddah (King Abdulaziz University Hospital) and Al-Khobar (King Fahd Hospital of the University). Samples were collected from various demographic groups and anatomical sources to ensure comprehensive coverage.

Different resistance mechanisms contribute to the rise in carbapenem resistance. The most concerning is the production of carbapenemase enzymes (Heras et al., 2015). Carbapenemases are classified into Ambler classes A (*e.g.*, KPC, GES), B (*e.g.*, NDM, VIM, IMP), and D (*e.g.*, OXA–48-like). The uniformly elevated MIC values for meropenem and predominance of high imipenem MICs (≥16 µg/mL) further support the presence of potent carbapenemase activity among the studied isolates. The World Health Organization (WHO) reports a sharp rise in hospital-acquired KPC-producing *K. pneumoniae*, a serious concern as KPCs are resistant to last-line antimicrobials (Pitout, Nordmann & Poirel, 2015). This study found that regional differences of Al-Khobar isolates showed KPC (class A) and OXA–48 (class D), while Jeddah isolates showed KPC and IMI (class A), NDM and IMP (class B), and OXA–48 (class D).

The main concern is the higher co-occurrence of KPC and OXA-48 carbapenemase genes within the same *K. pneumoniae* strains. One of the most striking findings of this study is the exceptionally high rate (∼90%) of co-carriage of KPC and OXA-48 genes among isolates. Such a high prevalence of dual carbapenemase production is uncommon and may have profound clinical implications.

The coexistence of multiple carbapenemase determinants within the same isolate is often mediated by plasmids and other mobile genetic elements that facilitate horizontal gene transfer. The accumulation of class A (KPC) and class D (OXA-48-like) enzymes within a single strain may broaden β-lactam resistance profiles and complicate therapeutic management (Hansen, 2021; Karampatakis, Tsergouli & Behzadi, 2023). This phenomenon is often mediated by plasmids and mobile genetic elements, facilitating horizontal gene transfer and rapid dissemination. The uniformly elevated MICs for meropenem and the predominance of high imipenem MICs (≥16 µg/mL) further support the presence of potent carbapenemase activity among the studied isolates.

Reports from the Gulf region have described the circulation of OXA-48 and other carbapenemase-producing *K. pneumoniae* strains, suggesting active regional spread and genetic exchange (Heras et al., 2015; Shibl et al., 2013; Zowawi et al., 2014). Such emergence of strains carrying multiple resistance mechanisms likely reflects ongoing antimicrobial selective pressure and the evolution of highly resistant lineages (Lan et al., 2021).

Travelers, particularly during Hajj and Umrah, may contribute to the spread of NDM and OXA–48 carbapenemases in Saudi Arabia (Nordmann, Cuzon & Naas, 2009). Despite NDM originating from New Delhi, its increasing detection in Saudi Arabia suggests global dissemination. International travel and hospitalization abroad have also played a role (Khdary et al., 2020; Memish et al., 2015). This study also identified the presence of other carbapenemase genes, such as IMI, indicating the emergence of new variants. Despite the role of international travel, antibiotic misuse and poor hospital practices remain the primary drivers of resistance (Indrajith et al., 2021).

Early detection is essential to prevent the spread of KPC-producing bacteria, especially in critical care settings. Clinicians rely on polymyxins and tigecycline to manage KPC-producing infections (Sonnevend et al., 2015), and although carbapenems are less effective, high-dose regimens may still be useful (Mathers et al., 2013). Polymyxins, used strategically in combination therapy, remain active against KPCs (Al-Qadheeb et al., 2010). In Greece, 88% of patients were successfully treated for KPC-2 infection using combination therapy (Sakka et al., 2007). Tigecycline is also effective against multidrug-resistant Gram-negative bacteria (Yaghoubi et al., 2022).

Antimicrobial-susceptibility testing showed all isolates from Jeddah and Al-Khobar were resistant to at least one carbapenem, confirming the threat posed by MDR *K. pneumoniae*. Genotypic profiling is critical to guide intervention strategies. Techniques like ERIC-PCR provide insights into strain differentiation (Vaara, 2019), and the ERIC sequences used are mobile elements called MITEs (Maltezou, 2009). These incomplete palindromes are located near intergenic regions (De Azevedo Ramos et al., 2023). ERIC-PCR analysis revealed 12 distinct clusters at a 95% similarity threshold, indicating substantial genetic diversity among the isolates rather than limited variability. This suggests the presence of multiple circulating lineages across the studied regions, rather than the dominance of a single clonal outbreak. In light of the emerging convergence between hypervirulence and multidrug resistance, our findings should be interpreted beyond traditional phenotypic classifications. The observed strain clustering and resistance profiles may reflect the circulation of genetically related lineages with overlapping virulence and resistance determinants, rather than distinct hypervirulent or multidrug-resistant populations.

The spread of carbapenemase-producing *Enterobacteriaceae* in Saudi Arabia is attributed to massive tourism and immigration during Hajj and Umrah, Antimicrobial misuse, and Transmission *via* contaminated food or water sources. Genotypic characterization helps trace infection patterns and understand the evolution of resistance.

This study was conducted during the COVID-19 pandemic, which should be considered a potential confounding factor when interpreting the findings. Changes in hospital admission patterns, antimicrobial prescribing practices, infection control measures, and laboratory workflows during the pandemic may have influenced the observed prevalence, resistance profiles, and clonal distribution of CRKP isolates. Consequently, the results may not fully reflect transmission dynamics outside the pandemic context, and caution is warranted when extrapolating these findings to non-pandemic periods.

## Conclusion

Strengthening molecular surveillance and routine genotypic characterization of carbapenem-resistant *K. pneumoniae* is essential for early detection, outbreak prevention, and informed clinical decision-making at both the hospital and region. In this study, KPC and OXA-48 were highly prevalent among isolates from both regions, whereas NDM was detected only in isolates from Jeddah and not in Al-Khobar. However, this study lacked patient genetic background data and detailed DNA fingerprinting; the clonal variability in *K. pneumoniae* identified using ERIC-PCR provided valuable phylogenetic information. Hence, the observation should be interpreted cautiously due to the limited sample size from only two regions of Saudi Arabia. The high co-occurrence of KPC and OXA-48 within the same isolates represents a significant clinical concern and warrants further investigation. Also, the use of whole-genome sequencing (WGS), which the present study lacked, can provide greater discriminatory power than ERIC-PCR. A multi-faceted approach, including early screening protocols and detailed genetic profiling, is essential for identifying KPCs before widespread outbreaks. This will support the development of effective multidrug combinatorial therapies, particularly against hospital-acquired infections.

##  Supplemental Information

10.7717/peerj.21653/supp-1 Supplemental Information 1 Representative agarose gel electrophoresis showing PCR amplification of the OXA-48 gene in the tested bacterial isolates Lane M: 100 bp DNA ladder; Lane +Ve: positive control; Lane –Ve: negative control; Lanes 1–17: clinical bacterial isolates screened for the OXA-48 gene.

10.7717/peerj.21653/supp-2 Supplemental Information 2 Agarose gel image showing PCR amplification of carbapenemase genes in *Klebsiella pneumoniae* Lane M: 100 bp DNA ladder; Lane numbers indicate the *Klebsiella pneumoniae* isolates screened for carbapenemase genes.

10.7717/peerj.21653/supp-3 Supplemental Information 3 Raw data

10.7717/peerj.21653/supp-4 Supplemental Information 4 Uncropped dendrogram output from ERIC-PCR analysis of Klebsiella pneumoniae isolates This corresponds to the cropped dendrogram shown in Figure 2 of the main manuscript.

10.7717/peerj.21653/supp-5 Supplemental Information 5 Uncropped gel documentation images supporting PCR-based detection of carbapenemase genes

10.7717/peerj.21653/supp-6 Supplemental Information 6 Original gel images and electrophoresis documentation system outputs for representative Klebsiella pneumoniae isolates, included for transparency and reproducibility

## Abbreviations

AMR: Antimicrobial Resistance
CPE: Carbapenemase-Producing Enterobacteriaceae
CFU: Colony Forming Unit
CLSI: Clinical and Laboratory Standards Institute
CRKP: Carbapenem-resistant *Klebsiella pneumoniae*
DNA: Deoxyribonucleic Acid
ERIC-PCR: Enterobacterial Repetitive Intergenic Consensus Polymerase Chain Reaction
GIM: German Imipenemase
IMI: Imipenem-Hydrolyzing β-lactamase
IMP: Imipenemase
KAUH: King Abdulaziz University Hospital
KFHU: King Fahd Hospital of the University
KPC: *Klebsiella pneumoniae* carbapenemase
LB: Luria Bertani
MDR: Multidrug-Resistant
MIC: Minimum Inhibitory Concentration
NDM: New Delhi Metallo-β-lactamase
OXA–48: Oxacillinase-48
PCR: Polymerase Chain Reaction
SIM: Seoul Imipenemase
SME: *Serratia marcescens* Enzyme
SPM: São Paulo Metallo-β-lactamase
SPSS: Statistical Package for the Social Sciences
VIM: Verona Integron-Encoded Metallo-β-lactamase
WHO: World Health Organization
XDR: Extensively Drug-Resistant

## Additional Information and Declarations

### Data Availability

The following information was supplied regarding data availability: The raw data are available in the Supplemental Files.
