# Supplementary material for: Molecular characterization of Klebsiella pneumoniae isolated from different clinical sources in Eastern and Western Regions of Saudi Arabia Tertiary Hospitals
Source: PeerJ. 2026 Sep 2;14:e21653. doi: 10.7717/peerj.21653 (PMC13546626; doi:10.7717/peerj.21653)
Supplement: Supplementary file 1 [file peerj-14-21653-s004.docx]

**Supplementary Table S1. List of oligonucleotides used in the study for screening different classes of carbapenemases from clinical isolates.**

| **Sr. No.** | **Targeted gene** | **Sequence (5` to 3` Direction)** | Amplicon size (bp) | References |
| --- | --- | --- | --- | --- |
| 1 | KPC–F | TGTTGCTGAAGGAGTTGGGC | 270 | (1) |
| 2 | KPC–R | ACGACGGCATAGTCATTTGC |  |  |
| 3 | GES–F | ATGCGCTTCATTCACGCAC | 844 | (2) |
| 4 | GES–R | CTATTTGTCCGTGCTCAGG |  |  |
| 5 | NMC–F | GCATTGATATACCTTTAGCAGAGA | 2,158 | (3) |
| 6 | NMC–R | CGGTGATTAAAATCACACTGAGCATA |  |  |
| 7 | IMI–F | CTACGCTTTAGACACTGGC | 481 | (1) |
| 8 | IMI–R | AGGTTTCCTTTTCACGCTCA |  |  |
| 9 | SME–F | ACTTTGATGGGAGGATTGGC | 551 | (4) |
| 10 | SME–R | ACGAATTCGAGCATCACCAG |  |  |
| 11 | IMP–F | GGAATAGAGTGGCTTAATCTC | 232 | (5) |
| 12 | IMP–R | GGTTTAAAAAACAACCACC |  |  |
| 13 | SIM–F | TACAAGGGATTCGGCATCG | 570 | (5) |
| 14 | SIM–R | TAATGGCCTGTTCCCATGTG |  |  |
| 15 | VIM–F | CGCGGAGATTGARAAGCAAA | 247 | (1) |
| 16 | VIM–R | CGCAGCACCRGGATAGAARA |  |  |
| 17 | NDM–F | GGTTTGGCGATCTGGTTTTC | 621 | (5) |
| 18 | NDM–R | CGGAATGGCTCATCACGATC |  |  |
| 19 | SPM–F | AAAATCTGGGTACGCAAACG | 271 | (5) |
| 20 | SPM–R | ACATTATCCGCTGGAACAGG |  |  |
| 21 | GIM–F | TCGACACACCTTGGTCTGAA | 477 | (1) |
| 22 | GIM–R | AACTTCCAACTTTGCCATGC |  |  |
| 23 | OXA–48–F | GCGTGGTTAAGGATGAACAC | 438 | (6) |
| 24 | OXA–48–R | CATCAAGTTCAACCCAACCG |  |  |

**References**

1. Zaman SB, Hussain MA, Nye R, Mehta V, Mamun KT, Hossain N. A Review on Antibiotic Resistance: Alarm Bells are Ringing. Cureus. 2017 June 28;9(6):e1403.
2. Labuschagne CDJ, Weldhagen GF, Ehlers MM, Dove MG. Emergence of class 1 integron-associated GES-5 and GES-5-like extended-spectrum β-lactamases in clinical isolates of *Pseudomonas aeruginosa* in South Africa. Int J Antimicrob Agents. 2008 June;31(6):527–30.
3. Jin C, Zhang J, Wang Q, Chen H, Wang X, Zhang Y, et al. Molecular Characterization of Carbapenem-Resistant Enterobacter cloacae in 11 Chinese Cities. Front Microbiol. 2018 July 17;9:1597.
4. Hong SS, Kim K, Huh JY, Jung B, Kang MS, Hong SG. Multiplex PCR for Rapid Detection of Genes Multiplex PCR for Rapid Detection of Genes Encoding Class A Carbapenemases. Ann Lab Med. 2012 Sept 1;32(5):359–61.
5. Dogonchi A, Ghaemi E, Ardebili A, Yazdansetad S, Pournajaf A. Metallo-β-lactamase-mediated resistance among clinical carbapenem-resistant *Pseudomonas aeruginosa* isolates in northern Iran: A potential threat to clinical therapeutics. Tzu Chi Med J. 2018;30(2):90.
6. Candan ED, Aksöz N. *Klebsiella pneumoniae*: characteristics of carbapenem resistance and virulence factors. Acta Biochim Pol. 2015;62(4):867–74.
