# Supplementary material for: Molecular characterization of Klebsiella pneumoniae isolated from different clinical sources in Eastern and Western Regions of Saudi Arabia Tertiary Hospitals
Source: PeerJ. 2026 Sep 2;14:e21653. doi: 10.7717/peerj.21653 (PMC13546626; doi:10.7717/peerj.21653)
Supplement: Supplementary file 2 [file peerj-14-21653-s005.docx]

**Supplementary Table S2. PCR protocol with respect to the primers used to amplify each marker gene encoding carbapenemases**

| **Genes** | **Initial denaturation** | **Cycles** | **Denaturation** | **Annealing** | **Extension** | **Final extension** |
| --- | --- | --- | --- | --- | --- | --- |
| KPC | 95 ºC/5 min | 35 | 95 ºC/1 min | 56 ºC/1 min | 72 ºC/1 min | 72 ºC/5 min |
| GES | 95 ºC/5 min | 35 | 95 ºC/45 sec | 55 ºC/45 sec | 72 ºC/3 min | 72 ºC/5 min |
| NMC | 94 ºC/5 min | 30 | 94 ºC/1 min | 55-58 ºC/45 sec | 72 ºC/1 min | 72 ºC/10 min |
| IMI | 95 ºC/5 min | 35 | 95 ºC/1 min | 58 ºC/1 min | 72 ºC/1 min | 72 ºC/5 min |
| SME | 94 ºC/5 min | 25 | 94 ºC/30 sec | 50 ºC/30 sec | 72 ºC/1 min | 72 ºC/7 min |
| IMP | 94 ºC/10 min | 36 | 94 ºC/30 sec | 52 ºC/40 sec | 72 ºC/50 sec | 72 ºC/5 min |
| SIM | 94 ºC/5 min | 36 | 94 ºC/30 sec | 52 ºC/40 sec | 72 ºC/50 sec | 72 ºC/5 min |
| VIM | 95 ºC/5 min | 35 | 95 ºC/1 min | 56 ºC/1 min | 72 ºC/1 min | 72 ºC/5 min |
| NDM | 94 ºC/10 min | 36 | 94 ºC/30 sec | 52 ºC/40 sec | 72 ºC/50 sec | 72 ºC/5 min |
| SPM | 94 ºC/5 min | 36 | 94 ºC/30 sec | 52 ºC/40 sec | 72 ºC/50 sec | 72 ºC/5 min |
| GIM | 94 ºC/5 min | 30 | 94 ºC/40 sec | 52 ºC/1 min | 72 ºC/2 min | 72 ºC/5 min |
| OXA–48 | 94 ºC/5 min | 35 | 95 ºC/1 min | 52 ºC/1 min | 72 ºC/1 min | 72 ºC/10 min |
