# Supplementary material for: Molecular characterization of Klebsiella pneumoniae isolated from different clinical sources in Eastern and Western Regions of Saudi Arabia Tertiary Hospitals
Source: PeerJ. 2026 Sep 2;14:e21653. doi: 10.7717/peerj.21653 (PMC13546626; doi:10.7717/peerj.21653)
Supplement: Supplementary file 3 [file peerj-14-21653-s006.docx]

**Supplementary Table S3. List of Antibacterial drugs subjected to Antimicrobial-susceptibility testing along with their index of susceptibility and resistance.**

| **Antimicrobials used** | **Susceptible(S)**  **Zone diameter, mm** | **Intermediate(I) Zone diameter, mm** | **Resistant(R) Zone diameter, mm** |
| --- | --- | --- | --- |
| Aztreonam (ATM) | 22 | 16–21 | 15 |
| Cefotaxime (CTX) | ≥ 22 | 16–21 | ≤ 15 |
| Ceftazidime (CAZ) | ≥ 21 | 18–20 | ≤ 17 |
| Ceftriaxone (CRO) | ≥ 23 | 20–22 | ≤ 19 |
| Cefepime (FEP) | ≥ 25 | 19–24 | ≤ 18 |
| Ciprofloxacin (CIP) | ≥ 21 | 16–20 | ≤ 15 |
| Norfloxacin (NOR) | 17 | 13–16 | 12 |
| Levofloxacin (LEV) | ≥ 17 | 14–16 | ≤ 13 |
| Nitrofurantoin (F) | 17 | 15–16 | 14 |
| Trimethoprim-sulfamethoxazole (SXT) | 16 | 11–15 | 10 |
| Ticarcillin (TIC) | 19 | 15–18 | 14 |
| Piperacillin-tazobactam (TZP) | 21 | 18–20 | 17 |
| Kanamycin (K) | ≥ 18 | 14–17 | ≤ 13 |
| gentamicin (CN) | ≥ 15 | 13–14 | ≤ 12 |
| Imipenem (IPM) | 23 | 20–22 | 19 |
